# Enhanced CO_2_ electroreduction *via* interaction of dangling S bonds and Co sites in cobalt phthalocyanine/ZnIn_2_S_4_ hybrids†
†Electronic supplementary information (ESI) available. See DOI: 10.1039/c8sc03986k


**DOI:** 10.1039/c8sc03986k

**Published:** 2018-11-26

**Authors:** Chunjun Chen, Xiaofu Sun, Dexin Yang, Lu Lu, Haihong Wu, Lirong Zheng, Pengfei An, Jing Zhang, Buxing Han

**Affiliations:** a Beijing National Laboratory for Molecular Sciences , CAS Key Laboratory of Colloid and Interface and Thermodynamics , CAS Research/Education Center for Excellence in Molecular Sciences , Institute of Chemistry , Chinese Academy of Sciences , Beijing 100190 , P. R. China . Email: hanbx@iccas.ac.cn ; Email: sunxiaofu@iccas.ac.cn; b University of Chinese Academy of Sciences , Beijing 100049 , China; c Shanghai Key Laboratory of Green Chemistry and Chemical Processes , School of Chemistry and Molecular Engineering , East China Normal University , Shanghai 200062 , China; d Institute of High Energy Physics , Chinese Academy of Sciences , Beijing 100049 , China

## Abstract

The efficient electrochemical reduction of CO_2_ to CO in aqueous electrolyte is very interesting. Due to the critical electron-transfer step during the activation of CO_2_, it is important to design efficient strategies to engineer the electronic properties of catalysts to improve the electrochemical performance. Herein cobalt phthalocyanine (CoPc) supported on ZnIn_2_S_4_ (ZIS) nanosheets was synthesized. It was found that the hybrids showed excellent performance for CO_2_ electroreduction to CO in aqueous solution. The faradaic efficiency, current density and mass activity could reach 93%, 8 mA cm^–2^ and 266 mA mg_(CoPc)_^–1^, respectively. Introduction of Zn-defects resulted in dangling S bonds in the ZIS support, which interacted with Co active sites of CoPc *via* strong Co–S interaction. Mechanistic studies revealed that the enhancement of CO production over CoPc by Co–S interaction originated from the eased CO_2_ activation.

## Abstract 2

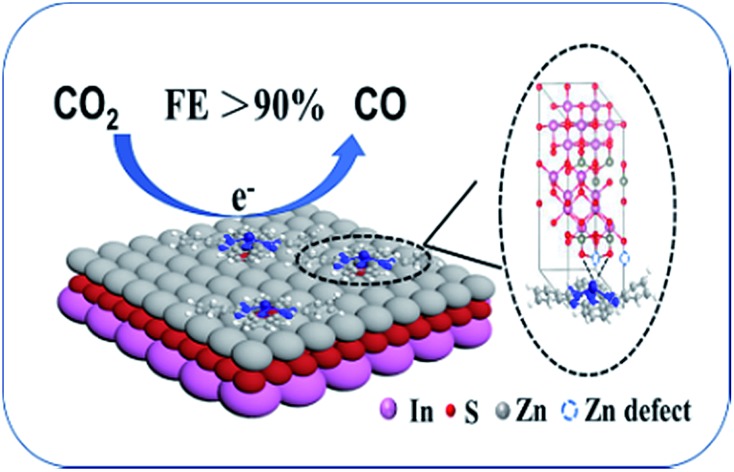
The strong Co–S interaction between CoPc and the dangling S bonds in CoPc/ZIS hybrids can enhance CO_2_ electroreduction to CO.

## Introduction

Global energy demand and climate change underpin broad interest in the sustainable reductive transformation of CO_2_ to value-added chemicals and fuels.^1^–^3^ Electrochemical reduction of CO_2_ is attracting increasing attention because of its potential for both the storage of intermittent electrical energy and controllable conversion into carbon-based products by the change of different parameters, such as electrode materials, electrolytes, and applied potentials.^4^–^7^ To date, a range of materials including metals, oxides, chalcogenides, metal–organic frameworks (MOFs), molecular complexes, and carbon-based materials have been explored as electrodes for catalyzing CO_2_ electroreduction.^8^–^19^ However, many of them still produce mixtures of products and require excessive overpotentials.

Metal phthalocyanines/porphyrins constitute an interesting class of materials with some obvious advantages, such as easy accessibility, chemical stability and structural tunability at the molecular level.^20^–^22^ It has been known that they can be used as electrocatalysts for CO_2_ reduction.^23^–^25^ Incorporation of cobalt porphyrin into covalent organic frameworks (COFs) can significantly improve their catalytic activity for reducing CO_2_ to CO, and they exhibited a faradaic efficiency (FE) of 90% together with an optimized initial turnover frequency as high as 3 s^–1^.^26^ In other cases, iron porphyrin, cobalt phthalocyanine (CoPc) and cobalt porphyrin were immobilized onto carbon nanotubes (CNTs),^27^–^29^ which can catalyze the electroreduction of CO_2_ to CO with remarkable activity, selectivity and durability in aqueous solution. However, the immobilization of metal phthalocyanines/porphyrins is achieved through π–π interaction between the macrocyclic complexes and the support, which is not a direct interaction between the catalytic sites and the supports.^26^,^28^ Because the metal centers in metal phthalocyanines/porphyrins are viewed as the catalytic sites in CO_2_ electroreduction,^27^,^30^ we believe that direct electronic interaction between the metal centers in phthalocyanines/porphyrins and the support would affect the catalytic performance for CO_2_ electroreduction more significantly.

As an important semiconductor material of ternary chalcogenides, ZnIn_2_S_4_ (ZIS) has attracted considerable attention because of its special electrical and optical properties.^31^,^32^ The defects of ZIS are easily formed, which could manipulate the energy band structure, carrier concentration, spin nature, and phonon vibration as well as migration.^33^ More importantly, the surface composition and electronic structure can be controlled by defects.^34^,^35^ It is noted that the catalytic activity of metal phthalocyanines/porphyrins is closely related to the surface composition and electronic structure of the supports.^24^ Therefore, ZIS can act as an excellent support to immobilize metal phthalocyanines/porphyrins and the interaction between the catalytic sites and supports can be tuned.

Herein, we developed an efficient strategy to facilitate CO_2_ electroreduction by immobilization of CoPc onto ZIS nanosheets. It was discovered that the FE of CO could reach 93% with a current density of 8 mA cm^–2^ and a mass activity of 266 mA mg_(CoPc)_^–1^ under optimal conditions. The high catalytic activity of CoPc/ZIS hybrids is attributed to the strong Co–S interaction between Co active sites and the dangling S bonds in the ZIS support. The strong Co–S interaction facilitated CO_2_ activation, leading to superior kinetics for CO production. As far as we know, this is the first study on the enhancement of CO_2_ electroreduction by Co–S interaction between the metal center in the macrocyclic complexes and the support. Besides, the Co–S interaction was studied in detail based on a series of control experiments.

## Results and discussion

**Fig. 1:**
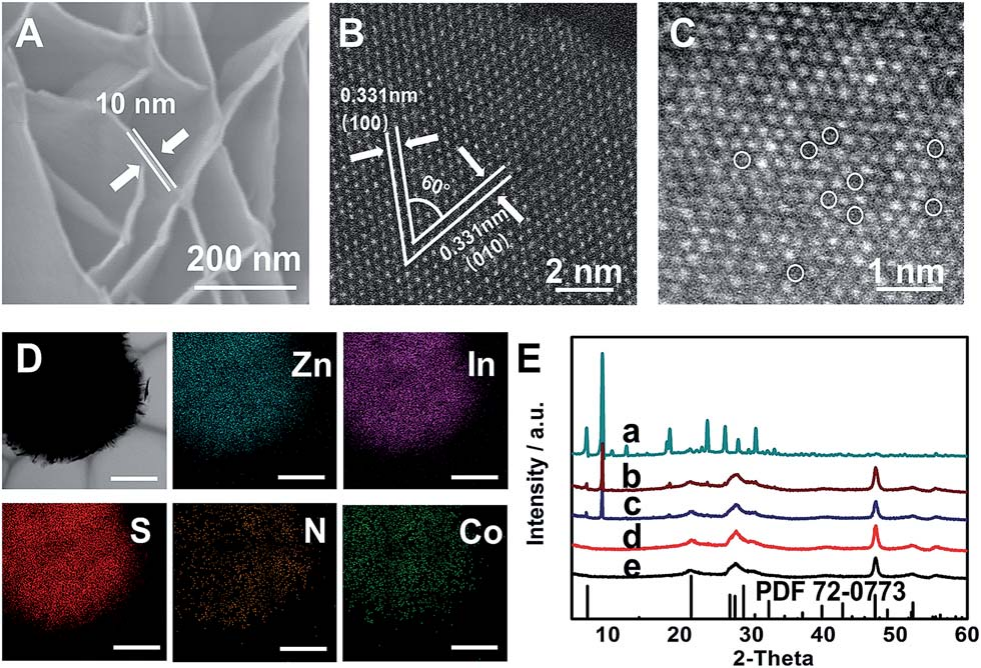
(A) SEM image of ZIS-200. (B and C) HAADF-STEM images of ZIS-200. (D) Elemental maps of 6.2-CoPc/ZIS-200 (the bar was 1 μm). (E) XRD spectra of the different samples: CoPc (a), 6.2-CoPc/ZIS-200 (b), 6.2-CoPc/ZIS-180 (c), ZIS-200 (d) and ZIS-180 (e). The XRD peaks of ZIS-180 and ZIS-200 were in agreement with that of hexagonal ZIS (PDF 72-0773).

ZIS nanosheets were fabricated *via* a hydrothermal method (see the ESI† for details).^31^ Scanning electron microscopy (SEM) images show that the ZIS products obtained at 180 °C (ZIS-180) and 200 °C (ZIS-200) had a flower-like morphology and were composed of nanosheets with a thickness of 10 nm (Fig. 1A and S1†). The X-ray diffraction (XRD) peaks of ZIS-180 and ZIS-200 agreed with that of hexagonal ZIS, indicating that the crystal structure and morphology of the ZIS obtained under two different temperatures were similar. The XPS spectra in Fig. S2† demonstrate that the chemical states of Zn^2+^, In^3+^ and S^2–^ in the ZIS nanosheets obtained at different hydrothermal temperatures were the same.^36^ Both ZIS-180 and ZIS-200 had mesoporous structures, and the surface area (*S*_BET_) was 102 and 90 m^2^ g^–1^, respectively (Fig. S3 and S4†). Additionally, the CO_2_ adsorption capacities of the ZIS-180 and ZIS-200 were similar, which were 8.1 and 8.4 cm^3^ g^–1^, respectively (Fig. S5†). The electrical conductivities of ZIS-180 and ZIS-200 were 0.00021 and 0.00022 S cm^–1^, respectively. They are very similar, so the influence of the electrical conductivities on the CoPc activity for CO_2_ reduction can be neglected.

To further reveal the fine structure of the synthetic ZIS nanosheets, their HAADF-STEM image was directly utilized to provide the atom arrangements in the ZIS nanosheets (Fig. 1B). It reveals that ZIS-200 has an interplanar spacing of 0.331 nm and a dihedral angle of 60°. This corresponds to the (100) and (010) planes of hexagonal ZIS, respectively. In addition, abundant Zn defects were observed in the magnified image, as shown in Fig. 1C.^31^ ZIS-180 was also characterized by HAADF-STEM, and the Zn defects were also observed (Fig. S6†). However, the amount of Zn defects in ZIS-180 was less than that in ZIS-200.

CoPc/ZIS hybrids were prepared by blending CoPc and ZIS in *N*,*N*-dimethylformamide (DMF) assisted by ultrasound. For clarity, *x*-CoPc/ZIS-180 and *x*-CoPc/ZIS-200 refer to the hybrids, and *x* represents the content (wt%) of CoPc. The CoPc content in CoPc/ZIS hybrids under different conditions can be calculated from the Co content determined by inductively coupled plasma optical emission spectroscopy (ICP-OES). The SEM and TEM images (Fig. S7†) confirm that 6.2-CoPc/ZIS-200 had a sheet-like morphology with an average thickness of 10 nm. No aggregated CoPc particles can be observed. The composition of the hybrids was determined by energy dispersive X-ray spectroscopy, indicating the homogeneous distribution of Co, N, Zn, In and S in the obtained nanosheets (Fig. 1d).

Furthermore, X-ray photoelectron spectroscopy (XPS) was employed to study the surface chemical states of the Co species and the interaction between Co species and the ZIS support. As shown in Fig. S8,† compared to the Co 2p peak of pure CoPc (780.87 eV),^37^ the Co 2p peaks of 6.2-CoPc/ZIS-200 and 6.2-CoPc/ZIS-180 shifted to lower binding energies by 1.03 eV and 0.67 eV, respectively. The S 2p peaks of 6.2-CoPc/ZIS-200 and 6.2-CoPc/ZIS-180 shifted to higher binding energies by 0.2 eV and 0.11 eV, compared with the pure ZIS (Fig. S9†). These results were consistent with previous literature,^38^ indicating a strong interaction between the Co and S in CoPc/ZIS hybrids. The results also suggest that the interaction between CoPc and ZIS-200 is stronger than that between CoPc and ZIS-180. Besides, XRD and Raman spectra in Fig. 1E and S10† provide further evidence for the formation of CoPc/ZIS hybrids.

**Fig. 2:**
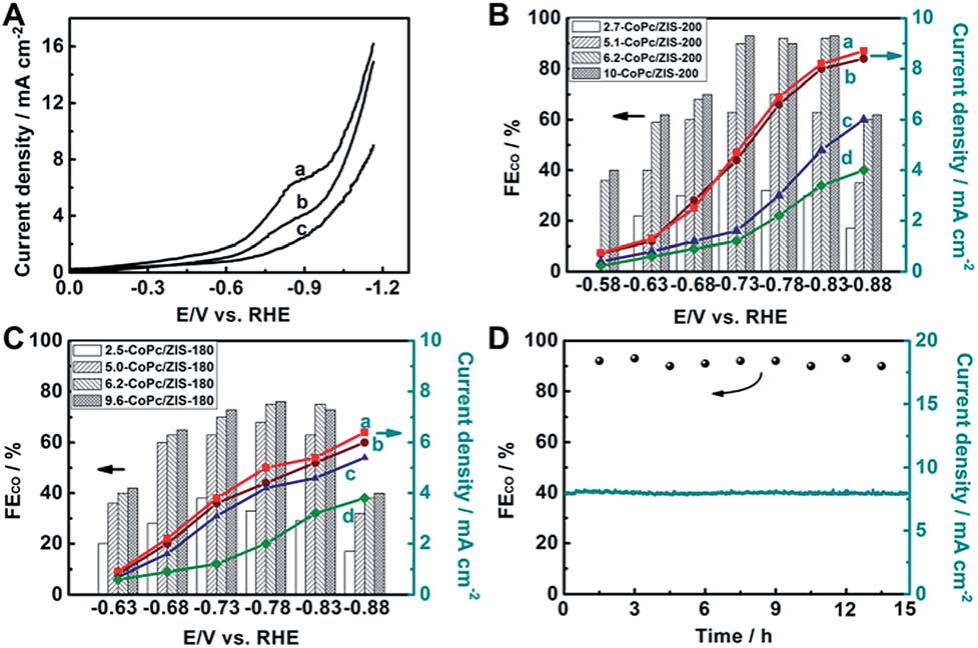
Electrocatalytic results in CO_2_-saturated 0.5 M KHCO_3_ electrolyte. (A) LSV curves of different electrodes: 6.2-CoPc/ZIS-200 (a), 6.2-CoPc/ZIS-180 (b) and CoPc (c). (B) FE(CO) and current density for CoPc/ZIS-200 at different potentials: 10-CoPc/ZIS-200 (a), 6.2-CoPc/ZIS-200 (b), 5.1-CoPc/ZIS-200 (c), and 2.7-CoPc/ZIS-200 (d). (C) FE(CO) and current density for CoPc/ZIS-180 at different potentials: 9.6-CoPc/ZIS-180 (a), 6.2-CoPc/ZIS-180 (b), 5.0-CoPc/ZIS-180 (c), and 2.5-CoPc/ZIS-180 (d). (D) Time curves of the electrolysis processes for 6.2-CoPc/ZIS-200 at –0.83 V *vs.* RHE and the gaseous products were quantified every 1.5 h.

To prepare the working electrode, we dispersed the hybrids in acetone with Nafion D-521 to form a homogeneous ink, and then spread them on carbon paper (CP). The performance of electroreduction of CO_2_ for the as-prepared electrodes was initially investigated using linear sweep voltammetry (LSV) in N_2_ or CO_2_ saturated 0.5 M KHCO_3_ aqueous solution with a standard three-electrode configuration. As illustrated in Fig. 2A, 6.2-CoPc/ZIS-200 showed better CO_2_ catalytic activity than CoPc/ZIS-180 and CoPc. A well-defined peak appears at around –0.84 V *versus* the reversible hydrogen electrode (RHE), suggesting the maximum CO_2_ reduction on the CoPc/ZIS catalyst. In the meantime, the onset potential with a significant current increase can be found in CO_2_ saturated 0.5 M KHCO_3_ aqueous solution as shown in Fig. S11,† indicating the reduction of CO_2_.

To further verify the occurrence of predominant CO_2_ reduction other than the H_2_ evolution reaction (HER), controlled potential electrolysis of CO_2_ was performed in a typical H-type cell to quantify the gas product by gas chromatography (GC) and the liquid product by nuclear magnetic resonance (NMR) spectroscopy, and the results are given in Fig. 2B and C and S12.† Only two products, CO and H_2_, were detected with a combined faradaic efficiency of around 100%. We found that with the increase of CoPc content, both the faradaic efficiency (FE) of CO and the current density over the CoPc/ZIS-200 and CoPc/ZIS-180 increased gradually at each given potential. When the CoPc content increased to 6.2 wt%, the FE(CO) and current density increased very slowly with further increase of the CoPc content. Therefore, we focus on 6.2-CoPc/ZIS hybrids in the following studies.

For 6.2-CoPc/ZIS-200, FE(CO) increased with increasing applied potential, and reached over 90% at –0.73 to –0.83 V *vs.* RHE. After that, FE(CO) decreased dramatically, indicating that the HER becomes the main reaction at a more negative applied potential. The maximum FE(CO) occurred at –0.83 V *vs.* RHE, and reached 93% with a 2-fold current density compared to that of CoPc. At this potential, the mass activity of 6.2-CoPc/ZIS-200 reached 266 mA mg_(CoPc)_^–1^, almost 40 times higher than that of CoPc (6.8 mA mg_(CoPc)_^–1^) under the same conditions. As can be seen from Table S1,† 6.2-CoPc/ZIS-200 has a comparable activity for CO_2_ reduction compared with other CoPc-based catalysts in previous literature. In contrast, the FE(CO) of 6.2-CoPc/ZIS-180 had the same trend, which was lower than that of 6.2-CoPc/ZIS-200, and the maximum FE(CO) only reached 73% at –0.83 V *vs.* RHE. Some additional experiments were performed to validate the performance of CoPc/ZIS. As shown in Fig. S13,† CoPc was directly loaded on CP, which showed a much lower FE(CO) and current density. Only H_2_ could be detected when ZIS was coated, suggesting that ZIS was not active in CO_2_ reduction. In order to eliminate the photocatalytic effect of ZIS, the electroreduction of CO_2_ over 6.2-CoPc/ZIS-200 was also conducted in the dark. The FE and current density of CO_2_ electroreduction in the dark were the same as that in light, indicating that the ZIS cannot be affected by light. Thus, the high catalyst efficiency originated from a synergistic effect of the catalyst and the support. Furthermore, a long-term operation was conducted at –0.83 V *vs.* RHE for 6.2-CoPc/ZIS-200 with a time of 15 h. The current density and the FE(CO) did not change with time during the entire period, as shown in Fig. 2D. This indicated that 6.2-CoPc/ZIS-200 was stable in the process of CO_2_ electroreduction.

**Fig. 3:**
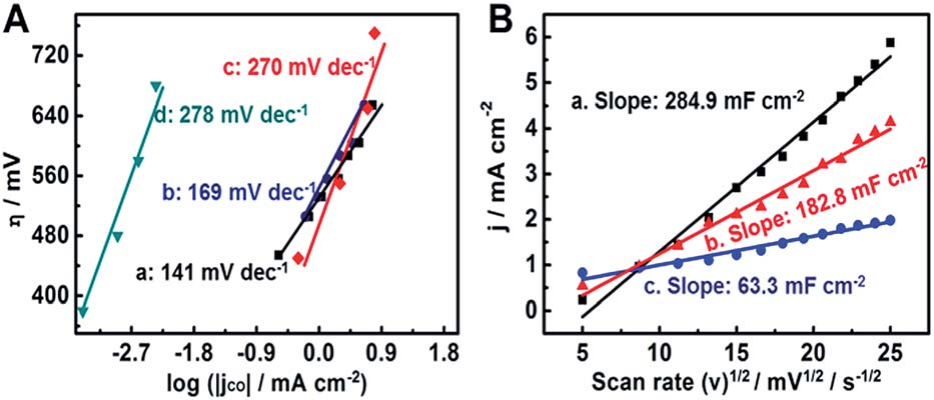
(A) Tafel plots for CO production over different electrodes: 6.2-CoPc/ZIS-200 (a), 6.2-CoPc/ZIS-180 (b), CoPc (c), and cobalt porphyrin (d). (B) The reduction current density at –0.83 V *vs.* RHE plotted against the square root of the scan rate for 6.2-CoPc/ZIS-200 (a), 6.2-CoPc/ZIS-180 (b) and CoPc(c).

In order to clarify the difference in catalytic activity for electrochemical CO_2_ reduction, we studied the Tafel plots of 6.2-CoPc/ZIS-200 and 6.2-CoPc/ZIS-180. As shown in Fig. 3A, the Tafel slopes for 6.2-CoPc/ZIS-200 and 6.2-CoPc/ZIS-180 were 141 mV dec^–1^ and 169 mV dec^–1^, respectively. They were much lower than those of analogous CoPc (270 mV dec^–1^)^39^ and cobalt porphyrin (278 mV dec^–1^).^26^ Moreover, the electrochemical activities of the different CoPc/ZIS electrodes were characterized by single-sweep polarography. According to the Randles–Sevcik equation, the reduction current density at –0.83 V *vs.* RHE plotted against the square root of the scan rate is shown in Fig. 3B. 6.2-CoPc/ZIS-200 had a larger slope than 6.2-CoPc/ZIS-180, leading to increased electrochemically active surface area and more catalytically active sites for CO_2_ electrochemical reduction. The results provide more evidence that 6.2-CoPc/ZIS-200 had a higher activity for reducing CO_2_ than 6.2-CoPc/ZIS-180, and the reasons will be discussed in the following sections.

**Fig. 4:**
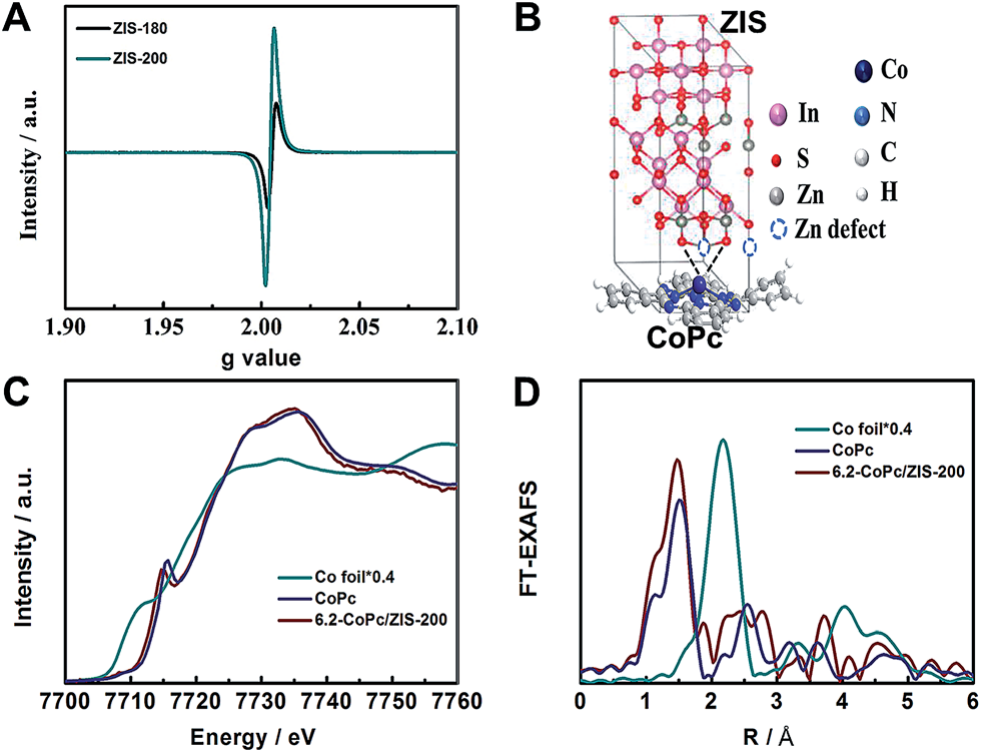
(A) EPR spectra of ZIS-200 and ZIS-180. (B) Schematic illustration of the dangling S bonds from Zn-defects which affected CoPc molecules *via* Co–S interactions. (C) XANES spectra at the Co K-edge. (D) Fourier-transformed Co K-edge EXAFS spectra.

The actual surface chemical states of Co atoms are strongly dependent on the surface states of ZIS, which would affect the catalytic activity. From Fig. 4A, it can be observed that ZIS-200 and ZIS-180 possessed a similar electron paramagnetic resonance (EPR) signal (*g* = 2.003), corresponding to the electrons captured by Zn-defects.^40^ The difference in the EPR signal intensity indicates that ZIS-200 had an obviously higher concentration of Zn-defects than ZIS-180. The formation of Zn-defects resulted in the dangling S bonds, which could accommodate electrons. The dangling S bonds can affect the Co centers of CoPc molecules directly *via* the strong Co–S interaction (Fig. 4B). In addition, ZIS-220 (obtained at 220 °C) was also studied to further compare the effect of Zn-defects. As shown in the EPR results in Fig. S14,† the concentration of Zn-defects in ZIS-220 was similar to that in ZIS-200. We also studied the CO_2_ electroreduction activity over CoPc/ZIS-220. The faradaic efficiency of CO and the total current density were 92.6% and 8.1 mA cm^–2^ at –0.83 V *vs.* RHE, respectively, which was similar to the results over 6.2-CoPc/ZIS-200. Combined with the results of 6.2-CoPc/ZIS-180, we can conclude that the CO_2_ electroreduction activity over CoPc/ZIS correlated with the concentration of Zn-defects.

In order to further clarify the Co–S interaction in 6.2-CoPc/ZIS-200, the X-ray absorption near-edge structure (XANES) spectra and Fourier-transformed Co K-edge extended X-ray absorption fine structure (EXAFS) spectra were recorded. As shown in Fig. 4C, the absorption edge position of Co for 6.2-CoPc/ZIS-200 was shifted to a lower energy compared with the pure CoPc, suggesting that the Co atoms exist in a lower oxidation state affected by the Co–S interaction. This transformation was also verified by the shift of the Co 2p XPS peak to lower binding energy (Fig. S8†). As shown in the Fourier transform of the EXAFS spectra of 6.2-CoPc/ZIS-200 (Fig. 4D), the peaks at around 1.6 Å and 1.9 Å can be attributed to the Co–N bond and the Co–S bond, respectively. The quantitative coordination configuration of the Co atom can be obtained by EXAFS fitting (Fig. S15 and Table S2†). These results indicate that Co–S interaction exists in 6.2-CoPc/ZIS-200. *In situ* XAFS was used to study the Co–S interaction during the electroreduction (Fig. S16†). The Co–S interaction can be observed at –0.83 V *vs.* RHE, as shown in Fig. S17.† This indicates that the Co–S interaction exists during the electroreduction.

Furthermore, we also used the zeta-potentials to verify the surface states of ZIS. From Fig. S18,† the zeta potentials for ZIS-200 and ZIS-180 were –35 mV and –20 mV, respectively. This demonstrates that negative charge exists on the surface of ZIS, and the negative charge increases with increasing Zn-defect concentrations. As the content of CoPc increased, the zeta-potential value gradually shifted to the positive side. It is worth noting that the zeta-potential values remained stable when a certain amount of CoPc was loaded on ZIS, indicating that the dangling S bonds have been fully covered by CoPc. As shown in Fig. 2B and C, a similar trend can be found between FE(CO) and the zeta-potential values when the content of CoPc changed. We can also find that ZIS-200 needs more CoPc to neutralize the negative charge, which proved the presence of more dangling S bonds in ZIS-200. This further provided evidence for enhancing CO_2_ electroreduction on CoPc/ZIS hybrids *via* Co–S interaction.

From the above results, we can draw a conclusion that the enhanced CO_2_ electroreduction on CoPc/ZIS hybrids was closely related to the Co–S interaction. According to previous literature on Co-based catalysts, Co^+^ is considered as the active site for CO_2_ reduction. Thus Co^2+^/Co^+^ redox transition is crucial for the activation of reduction of CO_2_ over CoPc/ZIS. As shown in the XPS Co 2p spectra and XANES spectra, the Co signal of 6.2-CoPc/ZIS-200 shifted to lower oxidation states (Fig. S8† and 4C). This indicated that the transition from Co^2+^ to Co^+^ over 6.2-CoPc/ZIS-200 became easier, which can facilitate CO_2_ reduction. This conclusion was also verified by LSV (Fig. S19†). Significant Co^2+^/Co^+^ redox transition can be observed at a more positive potential for 6.2-CoPc/ZIS-200 than CoPc. The positive shift of the Co^2+^/Co^+^ redox potential suggests more Co(i) sites in the 6.2-CoPc/ZIS-200 catalyst than CoPc at low overpotentials. Thus, we can conclude that the enhancement of CO_2_ reduction over CoPc/ZIS hybrids results from the easier Co^2+^/Co^+^ redox transition, which was achieved by the Co–S interaction.

## Conclusions

In summary, we synthesized CoPc/ZIS hybrids as efficient catalysts for CO_2_ electrochemical reduction to CO in aqueous electrolyte. The amount of Zn-defects could be controlled by varying the hydrothermal temperature for the synthesis of ZIS nanosheets, which provided dangling S bonds to interact with CoPc molecules. For the CO_2_ electrochemical reduction, 6.2-CoPc/ZIS-200 hybrids exhibited a FE of 93% for CO production with a current density of up to 8 mA cm^–2^ and a mass activity of up to 266 mA mg_(CoPc)_^–1^. The detailed study indicated that the excellent catalytic performance could be mainly attributed to the strong Co–S interaction between CoPc molecules and the dangling S bonds in the ZIS support. This work provides a protocol for enhancing the efficiency of CO_2_ electrochemical reduction by regulating the electronic interaction *via* dangling bonds.

## Conflicts of interest

There are no conflicts to declare.

## Supplementary Material

Supplementary information
