# Supplementary material for: Enhanced CO2 electroreduction via interaction of dangling S bonds and Co sites in cobalt phthalocyanine/ZnIn2S4 hybrids
Source: Chem Sci. 2018 Nov 26;10(6):1659–63. doi: 10.1039/c8sc03986k (PMC6368213; doi:10.1039/c8sc03986k)
Supplement: Supplementary file 1 [file SC-010-C8SC03986K-s001.pdf]

Electronic Supplementary Material (ESI) for Chemical Science.
This journal is © The Royal Society of Chemistry 2018

Supporting Information

Enhanced CO_2 electroreduction via interaction of dangling S bond and Co sites in Cobalt phthalocyanine/$ZnIn_2S_4$ hybrids

Chunjun Chen,[a,b] Xiaofu Sun,[a,b]* Dexin Yang,[a,b] Lu Lu,[a,b] Haihong Wu,[c] Lirong Zheng,[d] Pengfei An,[d] Jing Zhang,[d] Buxing Han[a,b]*

[a]Beijing National Laboratory for Molecular Sciences, Key Laboratory of Colloid and Interface and Thermodynamics, Institute of Chemistry, Chinese Academy of Sciences, Beijing 100190, P. R. China

[b]School of Chemistry and Chemical Engineering, University of Chinese Academy of Sciences, Beijing 100049, China

[c]Shanghai Key Laboratory of Green Chemistry and Chemical Processes, School of Chemistry and Molecular Engineering, East China Normal University, Shanghai 200062, China

[d]Institute of High Energy Physics, Chinese Academy of Sciences, Beijing 100049, China

Experimental Section

Materials. $Zn(NO_3)_2\cdot6H_2O$, $In(NO_3)_3\cdot xH_2O$, L-cysteine and cobalt phthalocyanine (CoPc) were provided by J&K Scientific Ltd. Potassium bicarbonate, ethanol and acetone were obtained from Sinopharm Chem. Nafion N-117 membrane (0.180 mm thick, ≥ 0.90 meg/g exchange capacity), Nafion D-521 dispersion (5 % w/w in water and 1-propanol, ≥ 0.92 meg/g exchange capacity) and Toray Carbon Paper (CP, TGP-H-60, 19×19 cm) were purchased from Alfa Aesar China Co., Ltd. CO_2 (99.999 %) and N_2 (99.99 %) were provided by Beijing Analytical Instrument Company.

Synthesis of $ZnIn_2S_4$ (ZIS). The procedures were similar to that reported.[S1] In the experiment, 0.5 mmol $Zn(NO_3)_2\cdot6H_2O$, 1.0 mmol $In(NO_3)_3\cdot xH_2O$, 4 mmol L-cysteine and 60 mL distilled water were mixed under stirring. The mixed solution was poured into a Teflon vessel held in a stainless steel autoclave of 100 mL, which was maintained at 200 °C , 180 °C or 220 °C for 18 h. After the system was naturally cooled down to room temperature, the obtained black suspension was washed by ethanol and water for four times, and then dried in the vacuum freeze-drying equipment for further characterization.

Synthesis of CoPc/ZIS. 30 mg as-prepared ZIS was dispersed in 30 mL DMF with the assistance of sonication for 1 h. Then, a certain amount of CoPc in DMF was added to the ZIS suspension followed by 30 min of sonication to obtain a well-mixed suspension. The mixture was further stirred at room temperature for 24 h. Subsequently, the mixture was centrifuged and the precipitate was washed with DMF and ethanol for three times alternatively. Finally, the precipitate was dried at 60 °C under vacuum overnight to yield the final product. The CoPc/ZIS samples with various contents of CoPc were synthesized by the addition of various content of CoPc.

Materials characterizations. X-ray diffraction (XRD) analysis of the samples was performed on the X-ray diffractometer (Model D/MAX2500, Rigaka) with Cu-Kα radiation, and the scan speed was 5°/min. The morphologies of the as-synthesized materials were characterized by scanning electron microscopy (SEM) S-4800, transmission electron microscopy (TEM) JEOL-2100F and HAADF-STEM. The Co

loadings in the catalysts were determined by inductively coupled plasma optical emission spectroscopy (ICP-OES) method (VISTA-MPX). X-ray photoelectron spectroscopy (XPS) was performed on the Thermo Scientific ESCA Lab 250Xi using a 200 W monochromated Al Kα radiation. Zeta potential measurements in H_2O were performed on a Zetasizer Nano instrument (Malvern Instruments, Worcestershire, U.K.). The N_2 adsorption/desorption isotherms were determined using a Micromeritics ASAP 2020 sorptometer operated at 77 K, and then BET surface areas and pore volumes were obtained. The adsorption isotherms of CO_2 were determined at 298 K in the pressure range of 0-1 atm on a TriStar II 3020 device. Electron paramagnetic resonance (EPR) measurements of samples were carried out at room temperature on a JEOL E-500. X-ray absorption fine structure (XAFS) experiments were operated at the 1W1B, 1W2B beamline at Beijing Synchrotron Radiation Facility (BSRF). All collected spectra were analyzed using Athena and Artemis program within the Ifeffit package.

Electrochemical study. To prepare the CoPc/ZIS/CP electrode, 2 mg CoPc/ZIS prepared above were suspended in 1 mL actone with 50 μL Nafion D-521 dispersion (5 wt%) to form a homogeneous ink assisted by ultrasound. Then, 250 μL of the ink was spread onto the CP (1×1 cm^{-2}) surface by a micropipette and then dried under room temperature. The loading of CoPc/ZIS catalyst was 0.5 mg cm^{-2}.

All the experiments of CO_2 reduction were carried out on the electrochemical workstation (CHI 6081E, Shanghai CH Instruments Co., China). Linear sweep voltammetry (LSV) scans were conducted in a single compartment cell with a three electrodes configuration, which consisted of working electrode, a platinum gauze as counter electrode, and Ag/AgCl (saturated KCl solution) as reference electrode. Prior to experiment, the electrolyte was bubbled with N_2 or CO_2 at least 30 min to form N_2 or CO_2 saturated solution. LSV measurement in gas-saturated electrolytes was conducted in the potential range of 0.33 V to -0.97 V versus RHE at a desired 20 mV S^{-1} sweep rate. To obtain uniform electrolyte, slight magnetic stirring was employed in the process. All potentials cited in this work were referenced to the RHE. The potentials were converted to RHE using the formulas E (vs RHE) = E (vs Ag/AgCl) +

0.197 V + 0.0591 × pH.

The electrolysis experiments were conducted at 25 °C in a H-type cell[S2] with a working cathode, a counter anode (platinum gauze), and a reference electrode (Ag/AgCl with saturated KCl). In the experiment, Nafion-117 membrane was used as proton exchange membrane that separated the cathode and anode compartments. $KHCO_3$ aqueous solution (0.5 M) were used as electrolytes. In each experiment, the amount of electrolyte was 30 mL. Before starting the electrolysis experiment, the catholyte was bubbled with CO_2 for 30 min under stirring and the electrolysis was carried out under a steady stream of CO_2 (20 sccm).

Product analysis. The gaseous product of electrochemical experiments was collected using a gas bag and analyzed by gas chromatography (GC, HP 4890D), which was equipped with FID and TCD detectors using helium as the internal standard. The liquid product was analyzed by ^{1}H NMR (Bruker Avance III 400 HD spectrometer) in DMSO-d_6 with TMS as an internal standard.

Tafel analysis: The partial current densities for products under different potentials were measured. The overpotential was obtained from the difference between the equilibrium potential and the applied potential. Electrolysis experiments were performed at each potential to obtain the current density versus overpotential data in the H-type electrolysis cell as described above. Tafel plots were constructed from these data.

Electronic conductivity: A four-contact method was applied to measure the powder electronic conductivity of ZIS. The powder sample was pressed to disk at 4 MPa with two stainless-steel plungers, whose resistance was measured by a Keithley 2400 digital multimeter in fourwire mode. The conductivity of the sample was calculated according to the resistance and the size of the disk.

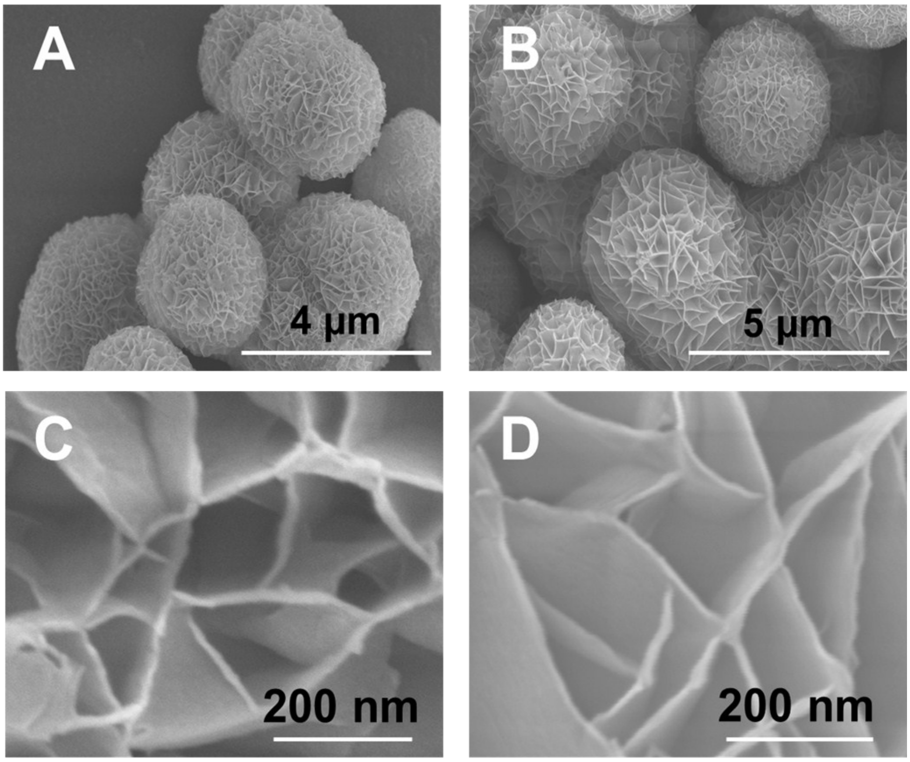

Figure S1. SEM images for ZIS: ZIS-200 (A, C); ZIS-180 (B, D).

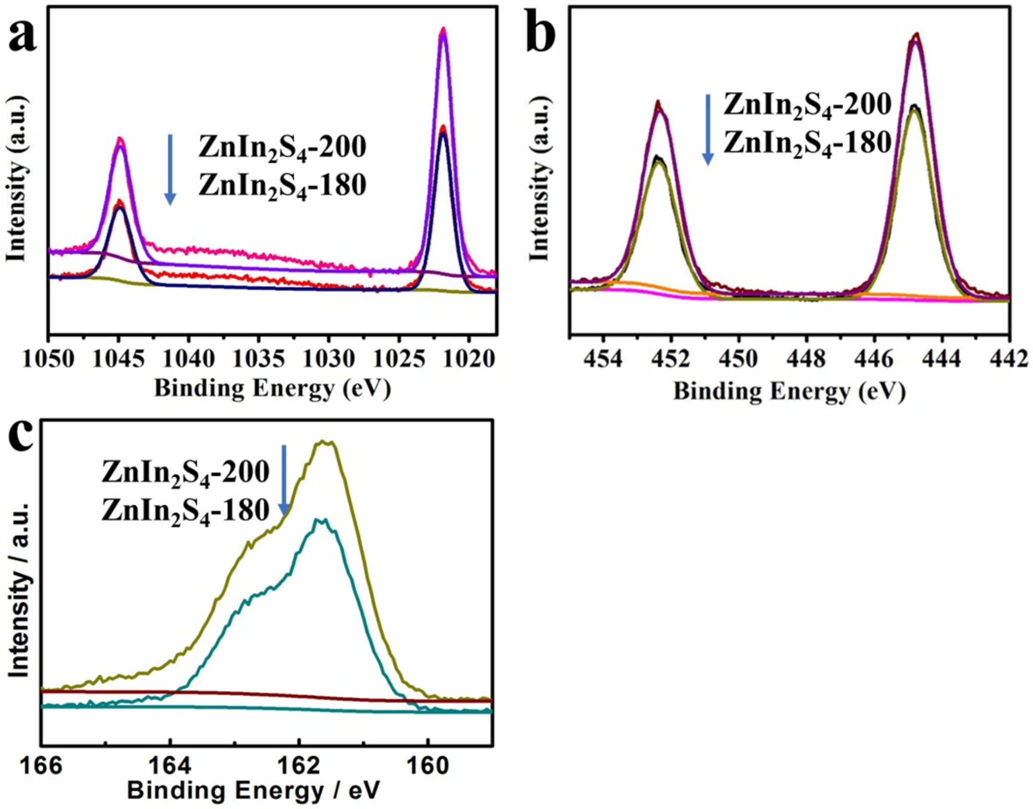

Figure S2. XPS spectra of ZIS: A) Zn element; B) In element; C) S element.

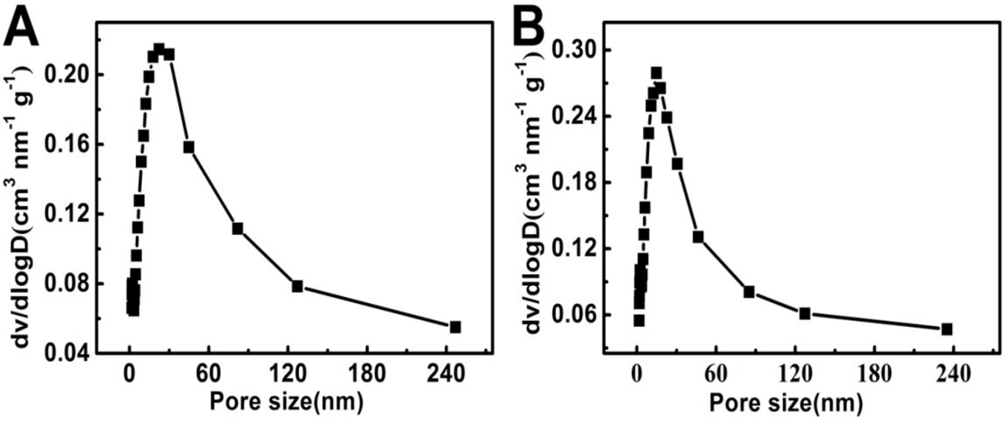

Figure S3. The pore size distribution of ZIS: A) ZIS-180; B) ZIS-200.

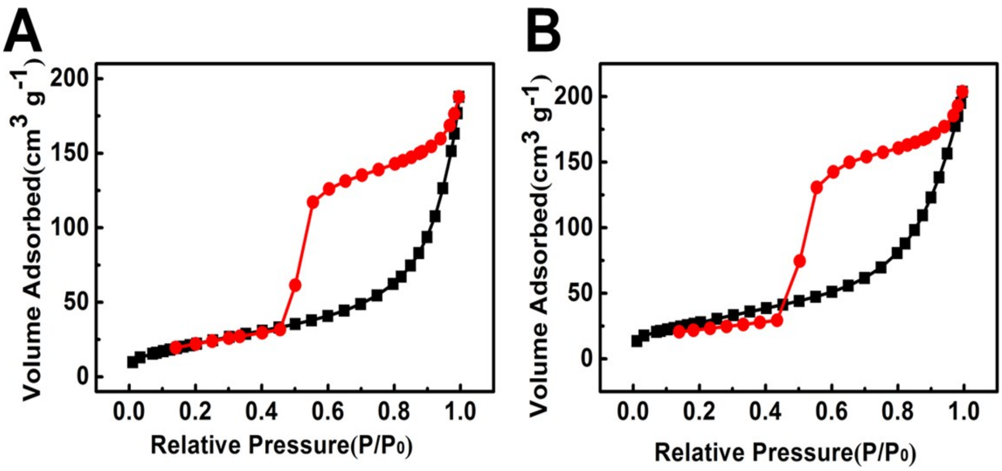

Figure S4. The N_2 adsorption-desorption isotherms of ZIS: A) ZIS-180; B) ZIS-200.

According to the literature, the surface area is mainly determined by the porous structures and the particle size. The different surface area for the ZIS-200 and ZIS-180 may be attributed to the slight difference of porous structures and the particle size. Therefore, it is not a positive correlation between SBET and the concentration of Zn-defects.

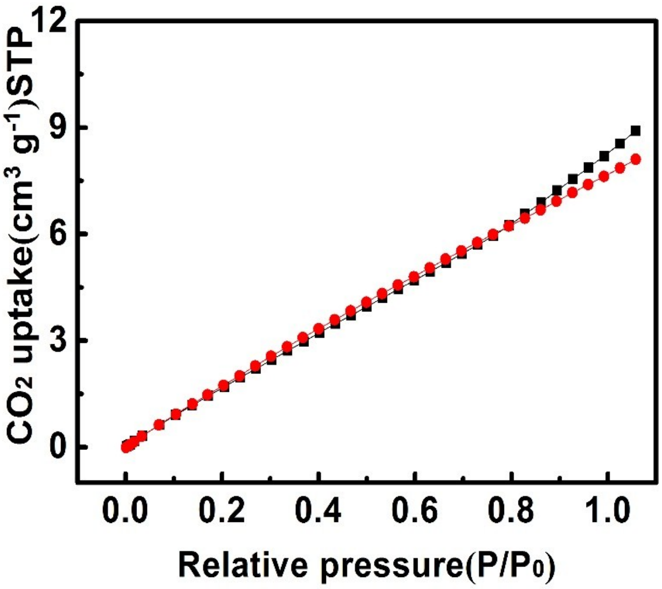

Figure S5. The CO_2 adsorption behaviors for ZIS: ZIS-180 (red); ZIS-200 (black)**.**

The CO_2 adsorption capacities not only depend on the surface area, but also depend on the interaction between the surface and CO_2. The CO_2 adsorption capacities of ZIS-200 is higher than that of ZIS-180, which can be explained the strong interaction between Zn-defects and CO_2.

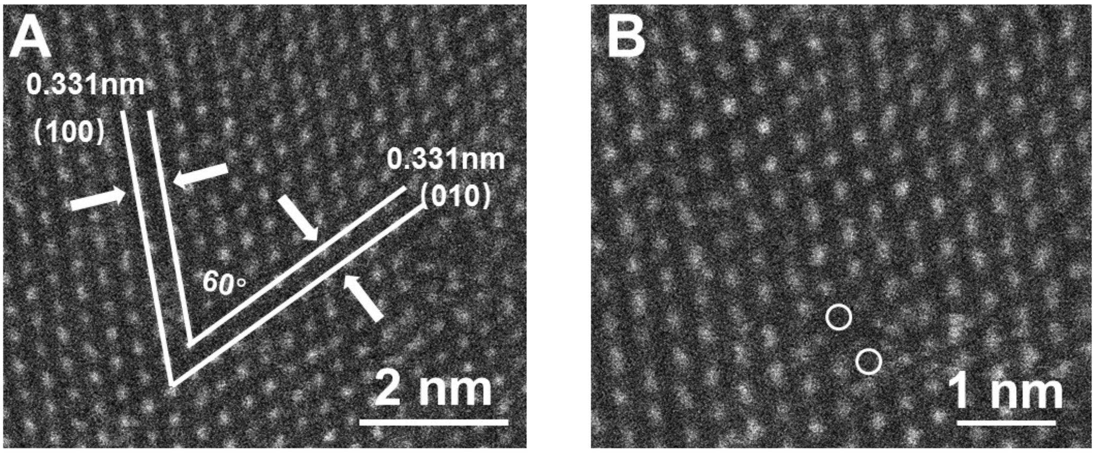

Figure S6. HAADF-STEM images of ZIS-180.

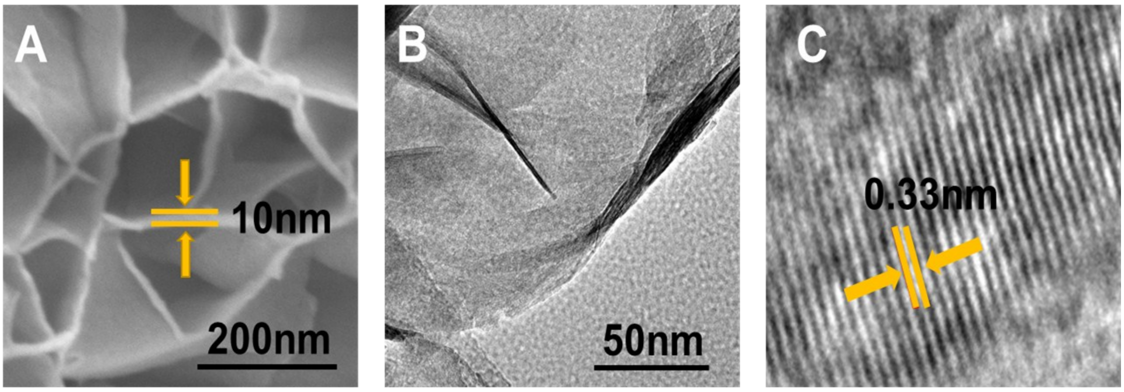

Figure S7. A) SEM image of 6.2-CoPc/ZIS-200. B-C) TEM image of 6.2-CoPc/ZIS-200.

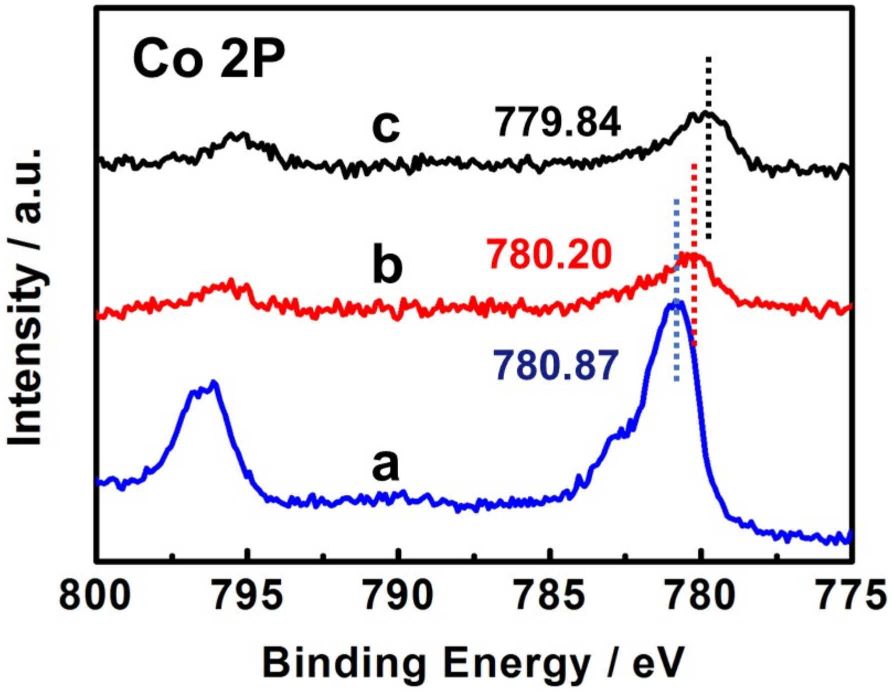

Figure S8. XPS spectra of Co: a pure CoPc; b 6.2-CoPc/ZIS-180 and c the 6.2-CoPc/ZIS-200.

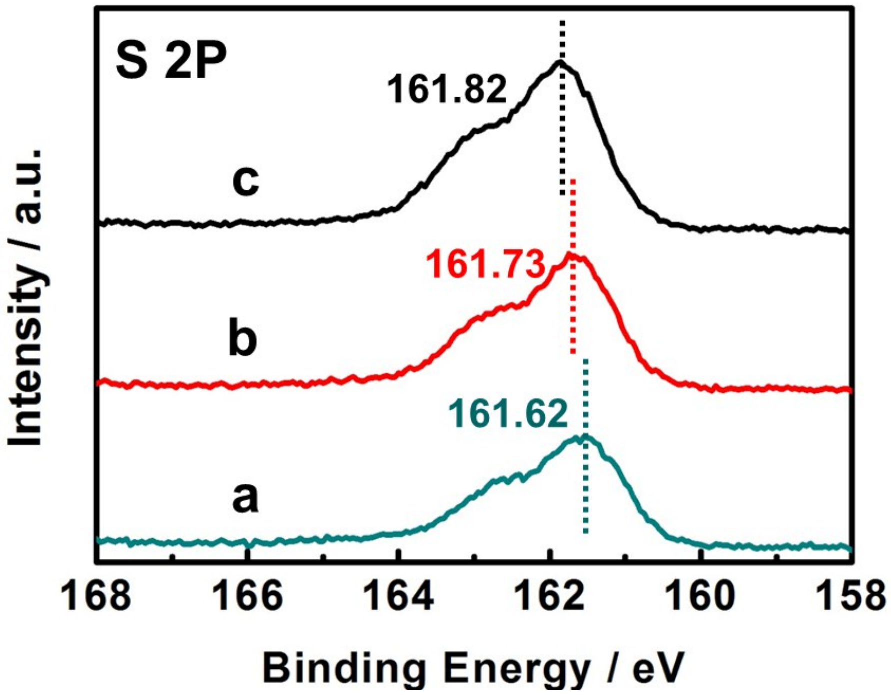

Figure S9. XPS spectra of S: a pure ZIS; b 6.2-CoPc/ZIS-180 and c the 6.2-CoPc/ZIS-200.

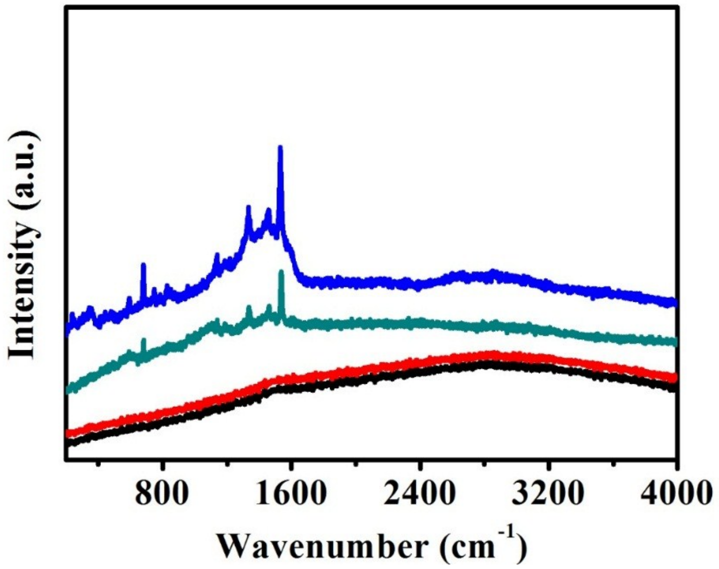

Figure S10. The Raman spectra of ZIS-180 (black); ZIS-200 (red); 6.2-CoPc/ZIS-180 (dark cyan) and 6.2-CoPc/ZIS-200 (blue).

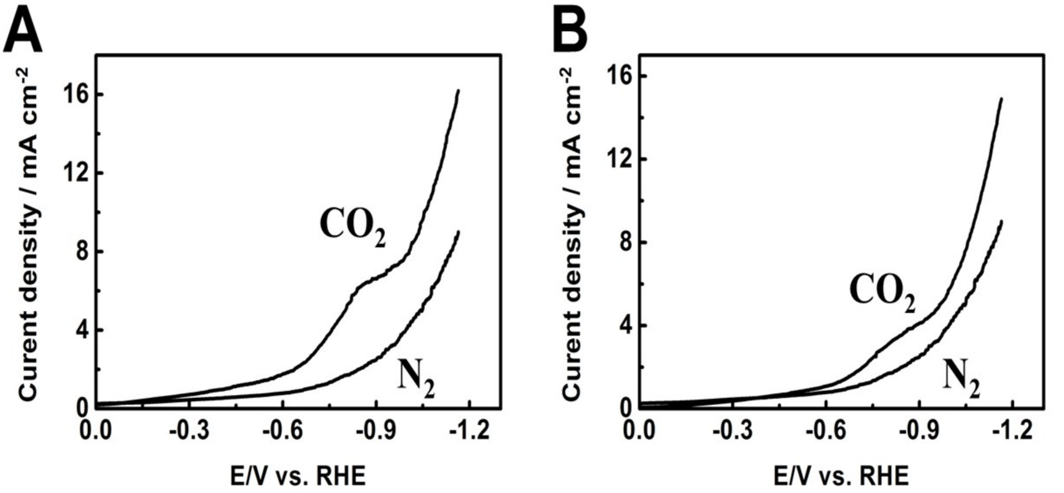

Figure S11. A) The LSV traces on 6.2-CoPc/ZIS-200; B) The LSV traces on 6.2-CoPc/ZIS-180.

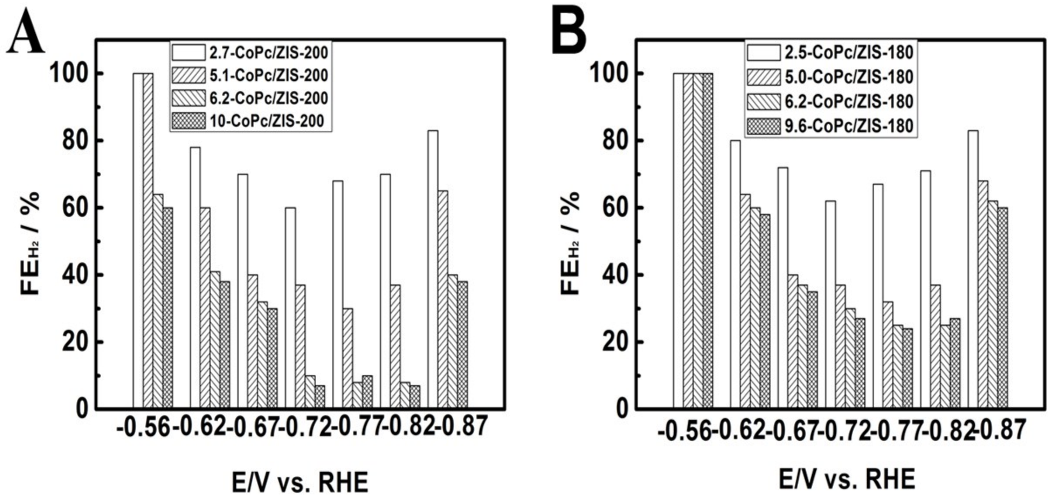

Figure S12. Faradaic efficiency for H_2 over CoPc/ZIS of different CoPc contents at various potentials: A) CoPc/ZIS-200; B) CoPc/ZIS-180.

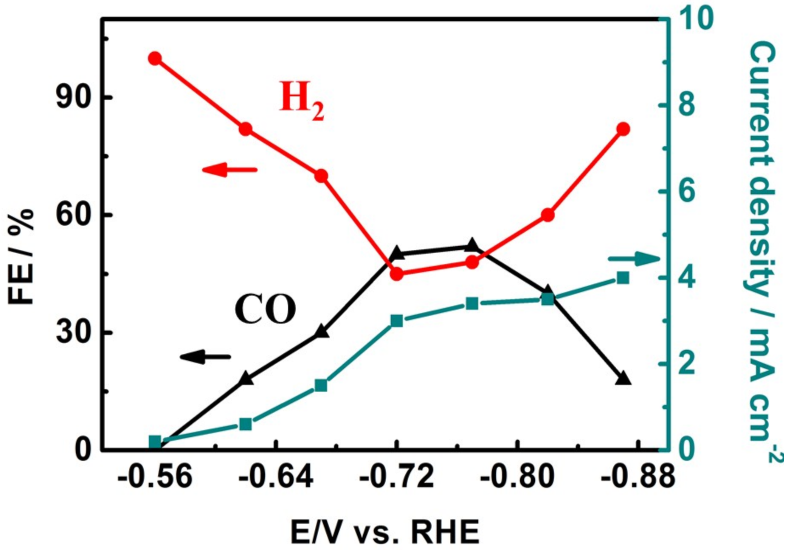

Figure S13. The Faradaic efficiency of CO and H_2 and current density over CoPc.

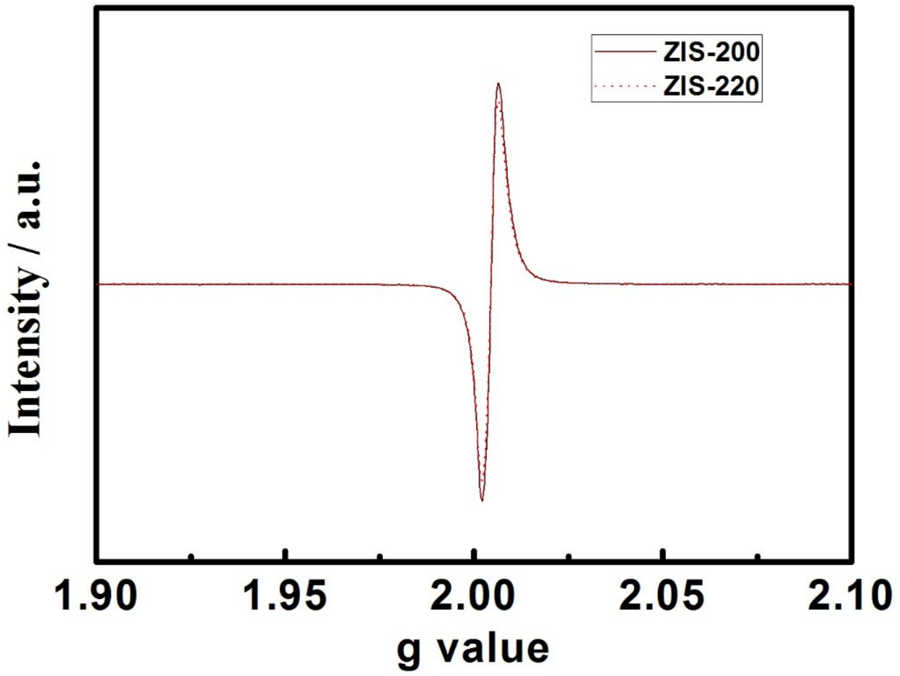

Figure S14. EPR spectra of ZIS-200 and ZIS-220.

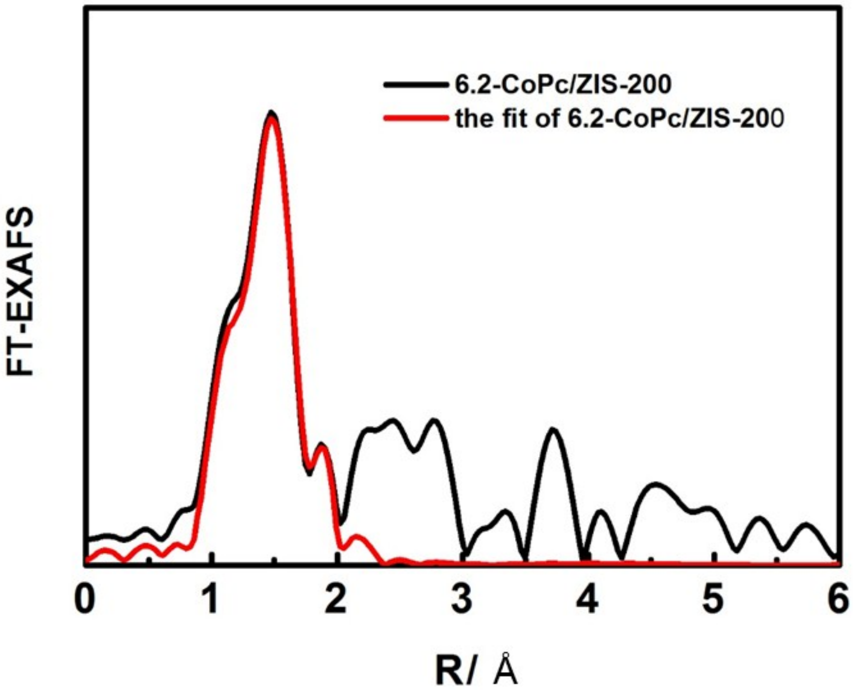

Figure S15. The EXAFS fitting curve of 6.2-CoPc/ZIS-200 composites.

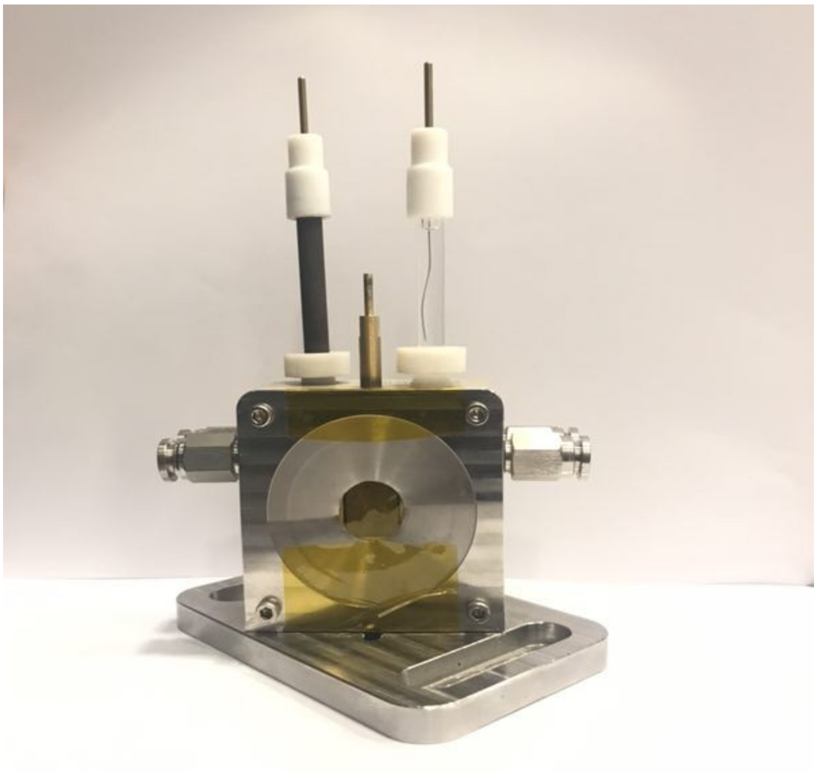

Figure S16. The in-stiu device of XAFS.

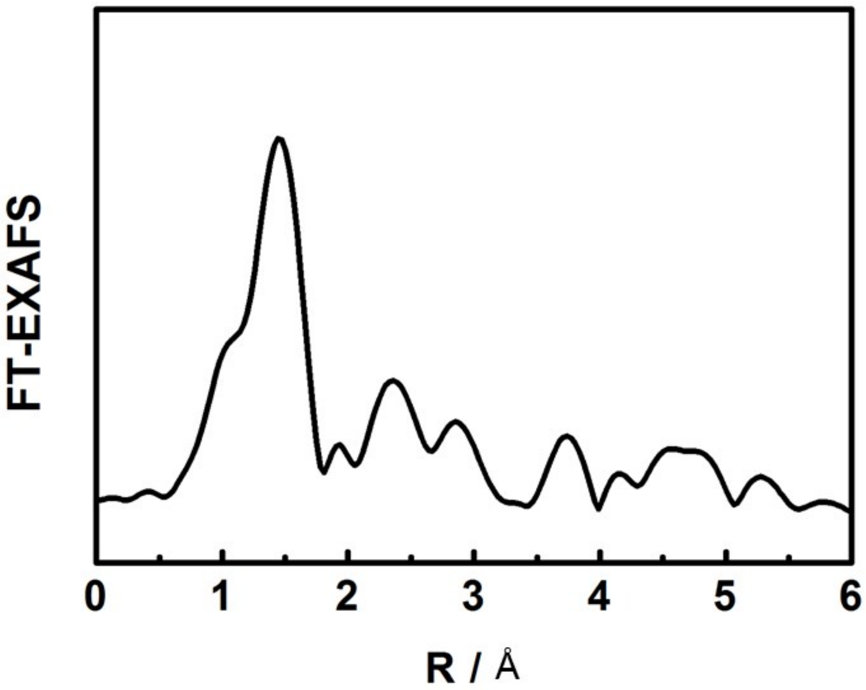

Figure S17. The Fourier transform of the EXAFS spectrum of 6.2-CoPc/ZIS-200 at -0.83V vs. RHE using the in-stiu device.

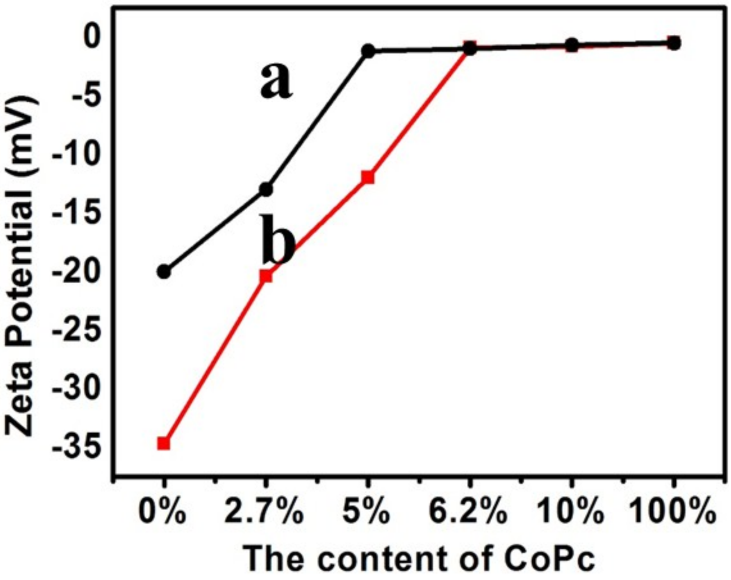

Figure S18. The zeta-potentials for CoPc/ZIS-180 (a) and CoPc/ZIS-200 (b) with different contents of CoPc.

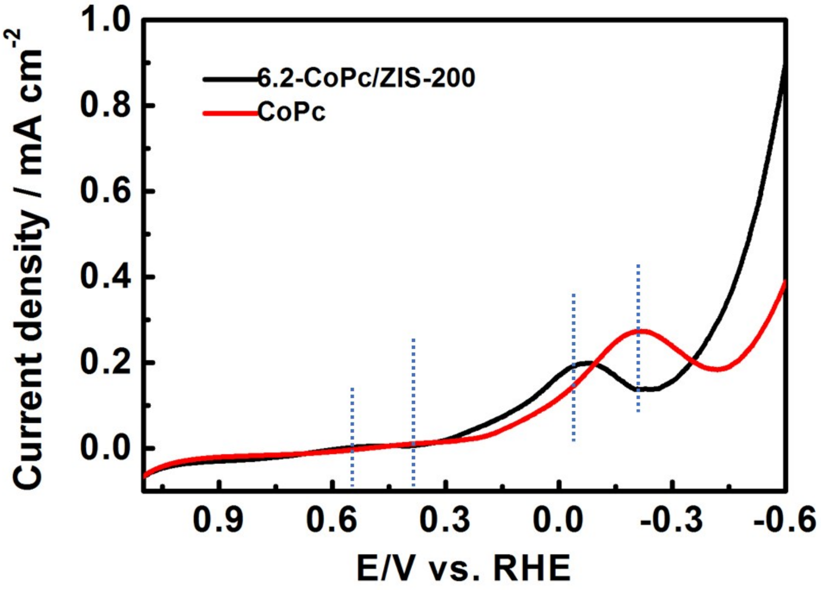

Figure S19. The LSV in 0.5 M aqueous $KHCO_3$ solution saturated with CO_2 at a scan rate of 10 mV s^{-1}.

Table S1 The summary of various CoPc-based electrodes used in CO_2 electroreduction

**Catalysts**	***j* (mA cm^{-2})**	**Electrode potential / V**	**Electrolyte**	**Faradaic efficiency / %**	**Ref.**
6.2-CoPc/ZIS-200	8	-0.83 V vs. RHE	0.5 M $KHCO_3$	93	This study
CoPc/CNT (2.5%)	10	-0.63 V vs. RHE	0.1 M $KHCO_3$	92	S3
Perfluorinated CoPc	4.4	-0.8 V vs. RHE	0.5 M $KHCO_3$	93	S4
CoPc-P4VP	2	-0.73 V vs. RHE	0.1 M NaH_2PO_4	89	S5
CoIIPc-tsGQwire	11.5	-2.1 V vs Ag/Ag^+	0.5M [Bmim]Tf_2N/MeCN	82.4	S6
CoPc-PVP	0.9	-1.6 V vs SCE	0.1 M NaH_2PO_4	84.1	S7
CoPc-graphite	0.5	-1.15 V vs SCE	0.05 M citrate buffer	60	S8

we have summarized various CO_2 electroreduction works in the literature over CoPc-based electrodes. As shown in Table S1, it can be seen that 6.2-CoPc/ZIS-200 composites have comparable activity for CO_2 reduction compared with other CoPc-based catalysts. Although the 6.2-CoPc/ZIS-200 was not the best catalyst for CO evolution, we think this study can offer a new way for enhancing the activity of CO_2 reduction.

Table S2 Structural parameters of 6.2-CoPc/ZIS-200 extracted from the EXAFS fitting. (S_0^2=0.80)

Sample	Scattering pair	CN	R(Å)	$\sigma^2(10^{-3}Å^2)$	ΔE_0(eV)	R factor
6.2-CoPc/ZIS-200	Co-N	4±0.9	1.90±0.02	8.5±1.1	4.6±1.2	0.0053
	Co-S	1.0±0.4	2.30±0.02	8.5±1.1	4.4±1.2	0.0054

S_0^2 is the amplitude reduction factor; CN is the coordination number; R is interatomic distance (the bond length between central atoms and surrounding coordination atoms); σ^2 is Debye-Waller factor (a measure of thermal and static disorder in absorber-scatterer distances); ΔE_0 is edge-energy shift (the difference between the zero kinetic energy value of the sample and that of the theoretical model). R factor is used to value the goodness of the fitting.

References

S1 X. Jiao, Z. Chen, X. Li, Y. Sun, S. Gao, W. Yan, C. Wang, Q. Zhang, Y. Lin, Y. Luo, Y. Xie, *J. Am. Chem. Soc.* **2017**, *139*, 7586-7594.

S2 X. Kang, Q. Zhu, X. Sun, J. Hu, J. Zhang, Z. Liu, B. Han, *Chem. Sci.* **2016**, *7*, 266-273.

S3 X. Zhang, Z. Wu, X. Zhang, L. Li, Y. Li, H. Xu, X. Li, X. Yu, Z. Zhang, Y. Liang, H. Wang, *Nat. Commun.* **2017**, *8*, 14675.

S4 N. Morlane´s, K. Takanabe, V. Rodionov, *ACS Catal* **2016**, *6*, 3092–3095.

S5 W. W. Kramer, C. C. L. McCrory, *Chem. Sci.* **2016**, *7*, 2506–2515.

S6 L. He, X. Sun, H. Zhang, F. Shao, *Angew. Chem. Int. Ed.* **2018**, *57*, 12453-12457.

S7 T. Yoshida, K. Kamato, M. Tsukamoto, T. Iida, D. Schlettwein, D. Wöhrle, M. Kaneko, *J. Electroanal. Chem.* **1995**, *385*, 209-225.

S8 C. M. Lieber, N. S. Lewis, *J. Am. Chem. Soc.* **1984**, *106*, 5033-5034
